# Cincho-Quinine

**Published:** 1873-04

**Authors:** 


*Cincho-Quinine.*

A medical friend who has tested the above article has drawn our attention to it as a preparation possessing all the virtues of the sulphate of quinine, without its intense bitterness or its tendency to produce deafness or cinchonism. It claims to combine all the alkaloids of Peruvian bark. It is virtually the bark in a shape for convenient administration. As such, it must contain important therapeutical properties not possessed by the sulphate. Most of our older practitioners regard the bark as superior to the sulphate as a tonic, and as more permanent in its antiperiodic properties. Its bulk and inconvenience of administration prevent its general use. The cincho-quinine, it is believed, will supply the desideratum resulting from our inability to use the crude bark.

J. R. Nicholls, of Boston, was the chemist who first succeeded in combining all the alkaloids of the bark into the beautiful white amorphus scales styled cincho-quinine. His successors—Billings, Clapp & Co., Boston, manufacturing chemists of high repute—continue to supply the trade with the quinine preparation. Its cost is but little over half the price of the sulphate.

